# Understanding the interplay of Caesarean delivery and genetic influences on intelligence and anxiety traits in offspring findings from genome-wide association studies

**DOI:** 10.1016/j.eurox.2025.100377

**Published:** 2025-03-05

**Authors:** Bita Fallahpour, Mahsa Danaei, Maryam Yeganegi, Fatemeh Jayervand, Sepideh Azizi, Heewa Rashnavadi, Seyed Alireza Dastgheib, Reza Bahrami, Amirhossein Shahbazi, Ali Masoudi, Kazem Aghili, Fatemeh Nematzadeh, Hossein Neamatzadeh

**Affiliations:** a Department of Psychiatry, Razi Hospital, University of Social Welfare and Rehabilitation Sciences, Tehran, Iran; b Department of Obstetrics and Gynecology, Iran University of Medical Sciences, Tehran, Iran; c Department of Obstetrics and Gynecology, Iranshahr University of Medical Sciences, Iranshahr, Iran; d Shahid Akbarabadi Clinical Research Development Unit, Iran University of Medical Sciences, Tehran, Iran; e Student Research Committee, Tehran University of Medical Sciences, Tehran, Iran; f Neonatal Research Center, Shiraz University of Medical Sciences, Shiraz, Iran; g Student Research Committee, Ilam University of Medical Sciences, Ilam, Iran; h Student Research Committee, Shahid Sadoughi University of Medical Sciences, Yazd, Iran; i Department of Radiology, Shahid Rahnamoun Hospital, Shahid Sadoughi University of Medical Sciences, Yazd, Iran; j Department of Education, Islamic Azad University, Shabestar Branch, Shabestar, Iran; k Mother and Newborn Health Research Center, Shahid Sadoughi University of Medical Sciences, Yazd, Iran

**Keywords:** Caesarean delivery, Intelligence, Anxiety, Genetic factors, GWAS, Offspring traits

## Abstract

**Background:**

Recent research suggests that genetic factors linked to Caesarean delivery may influence variations in children's intelligence and anxiety traits. This review synthesizes findings from genome-wide association studies (GWAS) to examine these associations, clarifying that it does not establish causation.

**Methods:**

This review systematically aggregated findings from GWAS studying the impact of Caesarean delivery on intelligence and anxiety traits. A thorough literature search was performed in key scientific databases like PubMed and Scopus, using various keywords related to delivery methods, cognitive traits, and psychological outcomes from 2005, when the first GWAS was published, through December 1, 2024. The inclusion criteria focused on original research articles published in English, excluding studies involving non-human subjects or without empirical data. The quality of the studies was assessed using a modified STROBE checklist adapted for GWAS.

**Results:**

Five GWAS identified 36 significant genetic loci associated with intelligence and anxiety traits in offspring related to Caesarean delivery. In terms of verbal intelligence, four alleles were found to be significantly linked to decreased scores, with allele rs1276529-G associated with a mean reduction of −2.04 units (p = 1E-6). Conversely, allele rs705670-G correlated with an increase in performance intelligence scores, resulting in a mean elevation of 2.3 units (p = 3E-7). Several alleles exhibited a negative correlation with overall intelligence, particularly rs17800861-A, which was associated with a mean decrease of 3.32 units (p = 7E-7). Significant risk alleles for anxiety were also identified, including rs62389045-C, linked to a 117 % increase in the risk of anxiety symptoms (p = 4E-8). Furthermore, in the context of self-injury, 17 risk alleles were identified, with allele rs117077436-C demonstrating an odds ratio of 11.34 (p = 3E-9).

**Conclusion:**

This study highlights multiple genetic loci associated with verbal performance, overall intelligence, and susceptibility to anxiety, revealing significant variations in offspring delivered via Caesarean section. While certain alleles are linked to increased risks of anxiety and self-injurious behavior, the results underscore the presence of genetic predispositions influencing cognitive and psychological outcomes. It is essential to emphasize that GWAS findings indicate associations rather than causal relationships. Further exploration into the biological mechanisms and environmental interactions that underlie these complex traits is warranted.

## Introduction

The global increase in Caesarean delivery rates, which have risen from approximately 7 % in 1990–21 % today, raises significant concerns about the implications for maternal and child health. Projections indicate that this rate could reach 28.5 % by 2030, resulting in around 38 million Caesarean deliveries annually [1], [2]. While Caesarean deliveries can reduce maternal and neonatal morbidity when medically justified, many are performed without clear indications, posing unnecessary risks [3], [4], [5]. The World Health Organization recommends an ideal cesarean rate of 10–15 %, suggesting that many regions exceed this threshold without corresponding improvements in health outcomes [6]. Research suggests that cesarean delivery may negatively affect children's cognitive development through direct and indirect pathways. Indirectly, cesarean births are linked to health issues like asthma, type I diabetes, and obesity, which can hinder cognitive functioning and academic performance [7], [8]. Directly, Caesarean deliveries can alter infants' gut microbiota, leading to lasting changes that may influence cognitive functions, mood, and stress responses [9], [10]. Emerging evidence points to disturbances in gut microbiota as potential contributors to cognitive disorders, such as autism spectrum disorder and attention deficit hyperactivity disorder (ADHD), underscoring the need to explore the long-term cognitive effects of cesarean delivery [11], [12]. While some studies indicate that cesarean-born children may have lower cognitive outcomes than those delivered vaginally, findings are mixed. For example, a study of 3666 Australian children revealed that cesarean-born children performed significantly worse on national numeracy tests at ages 8–9 [10]. A systematic review of seven studies found that four linked cesarean deliveries to reduced cognitive performance, while three did not, highlighting the need for further research to consider different cesarean types and control for confounding variables [8]. Additional findings show that cesarean-born children scored lower on cognitive tests at age five, particularly in visual-motor and intelligence assessments, and exhibited lower developmental scores in gross motor, fine motor, and language skills during early childhood. Longitudinal studies suggest that while cesarean-born children may initially have lower cognitive scores, these differences may diminish over time [7], [9]. However, inconsistencies remain regarding the causal relationship between cesarean birth and cognitive outcomes, with some studies finding no significant differences in developmental delays after controlling for confounding factors [10], [13].

Research is increasingly exploring the interplay between cesarean delivery and genetic influences on traits such as intelligence and anxiety [14], [15]. Studies indicate that cesarean-born children may exhibit lower cognitive abilities compared to those delivered vaginally, with large-scale research linking cesarean delivery to higher risks of ADHD and lower performance intelligence quotient (PIQ) scores [9], [16]. Furthermore, cesarean-born children often perform worse in national numeracy tests, highlighting potential impacts on cognitive development [8], [10]. Psychological implications are also significant, as evidenced by a study using UK Biobank data that found a correlation between cesarean birth and increased risks of anxiety and self-harm in adulthood, potentially exacerbated by genetic interactions [17]. Genome-wide analyses have identified specific loci associated with anxiety that may interact with the experience of cesarean birth [17], [18]. This systematic review aims to explore the association between cesarean delivery and cognitive and anxiety-related traits, focusing on the interplay between delivery mode and genetic influences identified through genome-wide association studies (GWAS). By synthesizing existing literature, the review seeks to clarify the effects of cesarean delivery on children's developmental outcomes, particularly regarding intelligence and psychological well-being. It will address inconsistencies in current research on the cognitive performance of cesarean-born children, examine the role of genetic factors, and provide insights into the long-term implications of cesarean births on cognitive and emotional development. Ultimately, this review aims to enhance understanding of how cesarean delivery may shape individuals' cognitive and psychological trajectories, informing clinical practices and public health policies to optimize maternal and child health.

## Materials and methods

### Search strategy

This review aims to synthesize and critically evaluate current findings from GWAS concerning the associations and interaction effects of Caesarean delivery on intelligence and anxiety traits. We employed a systematic approach to identify, evaluate, and summarize relevant literature, particularly focusing on studies that investigate the intricate interplay between genetic predispositions and environmental factors associated with the mode of delivery. A comprehensive literature search was conducted across major scientific databases, including PubMed, Scopus, Web of Science, and PsycINFO, as well as supplementary sources such as Google Scholar and relevant conference abstracts, ensuring a thorough collection of studies up to December 1, 2024. The search utilized a diverse array of keywords and combinations to encompass a wide range of related research, including terms such as "Caesarean delivery," "intelligence," "cognitive traits," "anxiety," "genome-wide association studies," "GWAS," "genetic epidemiology," "verbal intelligence quotient," "performance intelligence quotient," "full scale intelligence quotient," "self-harm," "interaction effects," "psychological assessment," "cognitive functioning," "obstetric outcomes," "mental health," "neurodevelopment," "child development," "risk factors," "longitudinal studies," "maternal health," "pediatric psychology," "quantitative analysis," "clinical outcomes," and "birth method impacts." This multifaceted strategy was designed to maximize the retrieval of studies pertinent to these topics. The search strategy was refined using Boolean operators such as AND, OR, and NOT, along with specific filters for publication types, study design, and age groups, ensuring a thorough examination of the literature. Moreover, reference lists from identified articles were meticulously reviewed to uncover any pertinent studies that may not have been included in the initial search, further enhancing the comprehensiveness of the findings.

### Inclusion and exclusion criteria

This review seeks to identify genetic variants associated with cognitive traits through a systematic evaluation of GWAS, which effectively reveal the genetic architecture of complex traits due to their large sample sizes and advanced statistical methods. These approaches improve the reliability of findings related to single nucleotide polymorphisms (SNPs) that may be missed by other research methods. While non-GWAS studies can offer valuable insights into cognitive symptoms and their genetic links, variability in methodologies can introduce confounding factors that obscure specific genetic connections. To ensure a focused analysis, the review employs strict inclusion and exclusion criteria. Inclusion criteria are: 1) studies must be original research published from 2005 onward to reflect current understanding and methodologies; 2) studies must use validated measures for cognitive assessment to ensure reliability; and 3) studies must involve human participants for direct applicability to real-world cognitive traits. Exclusion criteria include: 1) studies that do not prioritize genetic factors; 2) studies with limited longitudinal data; 3) studies with small sample sizes that may compromise robustness; and 4) unpublished and non-peer-reviewed studies to maintain scientific integrity. The review specifically examines genetic analyses related to cognitive traits in the context of various delivery methods, including planned and emergency Caesarean sections (CS) and vaginal deliveries. Future research should integrate findings from relevant non-GWAS studies to provide a broader perspective on the genetic influences on cognitive traits.

### Data extraction

Data extraction from each eligible study was carefully performed by two independent authors who collaborated to ensure accuracy and completeness. They used a standardized extraction form to capture essential information, including authors' names, publication years, sample sizes, and demographic characteristics of the study populations, such as reported traits, EFO traits, discovery sample ancestries, association counts, and a detailed sample description. Initially, both authors reviewed the studies independently and filled out the forms. They then met to compare their findings and address any discrepancies. For disagreements on specific data points or interpretations, they discussed the differences in an effort to reach a consensus based on the original studies. If they could not agree after thorough discussion, a third author was consulted. This third author provided an objective perspective, helping to resolve any ongoing discrepancies. This collaborative approach ensured that the extracted data was accurate and comprehensive, enhancing the validity of the reported findings in the study.

### Quality assessment

The quality assessment of GWAS was systematically conducted using a modified version of the Strengthening the Reporting of Observational Studies in Epidemiology (STROBE) checklist, specifically tailored for this context. This comprehensive evaluation considered multiple dimensions, including study design, population characteristics, genotyping methodologies, statistical analyses, and result reporting, with the aim of ensuring methodological rigor and transparency. Studies were categorized into high, medium, or low quality based on predefined thresholds: high-quality studies fulfilled all 12 criteria; medium-quality studies scored between 9 and 11; and those below 9 were classified as low quality. A specific scoring system was employed to quantitatively reflect adherence to the checklist criteria, while inter-rater reliability was rigorously assessed using Cohen's Kappa statistic to establish consistency among reviewers in the classification process. In cases involving more than two raters, Fleiss' Kappa was employed to gauge overall agreement, complemented by percentage agreement for a comprehensive evaluation of inter-rater reliability. Regular calibration meetings among reviewers facilitated discussions to align their interpretations of the criteria, enhancing classification consistency. Additionally, the evaluation underscored the importance of the robustness of statistical methods and the reproducibility of results as critical components in the overall quality assessment of the included studies.

### Statistical analysis

This review focused on qualitatively summarizing results from individual studies that explored the interaction effects between Caesarean delivery and genetic factors related to intelligence and anxiety traits, without conducting a meta-analysis. The findings were organized by identified genetic loci, discussing the potential functional roles of common genetic variants associated with these traits and the pathways involved in their development. A narrative synthesis approach was utilized to integrate results, categorizing them based on the associative relationships and interaction effects between Caesarean delivery and both intelligence and anxiety traits. This analysis highlighted trends in genetic findings related to different delivery methods while also considering demographic and environmental factors that could influence developmental outcomes. The overall goal of the synthesis was to elucidate patterns and establish connections between genetic predispositions and the observed traits in offspring.

### Ethical considerations

As this review synthesized existing studies and did not involve primary data collection from human participants, it did not require ethical approval. However, it acknowledged the importance of ethical considerations regarding patient consent and data handling in the original studies. By emphasizing adherence to ethical standards in GWAS research, particularly with vulnerable populations, the review-maintained transparency and integrity in reporting. It confirmed that all studies included adhered to ethical guidelines, with proper consent obtained from participants.

### Limitations of the review

This review has potential limitations, including inherent variability in study designs and methodologies across the included studies, which may impact the comparability of results. Differences in sample sizes, assessment tools, and definitions of intelligence and anxiety traits may lead to inconsistencies in findings. Moreover, the review is limited to published literature, which may introduce publication bias, as studies with null or negative results may be underrepresented. Future research should aim to address these limitations by conducting large-scale, well-designed studies that explore the multifaceted relationships between mode of delivery, genetic factors, and developmental outcomes. It is crucial to emphasize that while this review discusses associations identified in GWAS, these studies do not establish causality between Caesarean delivery and cognitive or anxiety traits.

## Results

### Selected studies characteristics

Table 1 presents an overview of five GWAS originating from two key publications that examine the interplay between genetic and environmental factors influencing cognitive and psychological traits. The studies by Smajlagić et al. [14] and Jia et al. [17] focus on intelligence and mental health, primarily analyzing data from individuals of European descent. Smajlagić et al. investigated dimensions of intelligence, including verbal intelligence quotient (VIQ), performance intelligence quotient (PIQ), and full-scale intelligence quotient (FSIQ), in relation to caesarean-section deliveries. Their discovery sample comprised 2421 individuals, including 1902 children. Conversely, Jia et al. explored the associations of anxiety and self-harm with caesarean-section births, utilizing significantly larger samples of 83,615 for anxiety and 96,462 for self-harm, primarily from British ancestry. Smajlagić et al. identified meaningful associations between cognitive abilities and early childhood stimuli within their child sample, while Jia et al. documented substantial genetic-environment interactions regarding anxiety and self-injurious behaviors among individuals born via caesarean delivery, based on an extensive dataset of 14,094 cases alongside control groups. The findings from these studies highlight a thorough investigation into the genetic and environmental influences on cognitive and psychological traits, with Smajlagić et al. reporting four associations related to intelligence, while Jia et al. examined up to 19 associations pertaining to self-harm.

**Table 1:** Characteristics of selected GWAS studies.

**First Author**	**Reported Trait**	**Discovery Sample Ancestry**	**Initial Sample Description**	**Association Count**
Smajlagić et al. [14]	CS, Verbal intelligence quotient	2421 European	1902 European ancestry child controls	4
Smajlagić et al. [14]	CS, Performance intelligence quotient	2421 European	1902 European ancestry child controls	2
Smajlagić et al. [14]	CS, Full scale intelligence quotient	2421 European	1902 European ancestry child controls	4
Jia et al. [17]	CS, Anxiety	83615 European	14,094 British ancestry cases not born by C-section,1788 British ancestry controls born by C-section,67,261 British ancestry controls not born by C-section	7
Jia et al. [17]	CS, Self-injurious behavior	96462 European	13,974 British ancestry cases not born by C-section,2167 British ancestry controls born by C-section,79,850 British ancestry controls not born by C-section	19

Abbreviations: CS - Caesarean Section; GWAS - Genome-Wide Association Studies; IQ - Intelligence Quotient.

### STROBE checklist

The scores for the studies were evaluated based on their alignment with specific items from the STROBE checklist, which assesses the clarity and comprehensiveness of results presentations. An analysis of the results sections in the studies by Smajlagić et al. [14] and Jia et al. [17] indicates a strong adherence to the STROBE checklist criteria. Both studies effectively present results illustrating the associations between caesarean-section delivery and anxiety and self-harm outcomes, complete with detailed reporting of odds ratios (ORs), confidence intervals (CIs), and p-values, thereby fulfilling STROBE item 20. However, both studies lack sufficient details regarding participant flow, as they do not specify the number of participants included or excluded from the analysis, which is mandated by STROBE item 14. While comprehensive descriptive data on participant characteristics are provided, this information is not incorporated within the results section, as per STROBE item 18. Nevertheless, both studies successfully present well-defined outcome data that meet the requirements of STROBE item 19, accurately describe main results with statistical significance in accordance with STROBE item 21, and include additional genome-wide by environment interaction analyses consistent with STROBE item 25. Inter-rater reliability in the evaluation of these studies was robust, demonstrated by a Cohen's Kappa statistic exceeding 0.75, indicating substantial agreement among reviewers on the scoring process. Periodic calibration meetings reinforced this alignment, ensuring that all reviewers interpreted checklist criteria consistently. Collectively, each study achieved a commendable score of 11 out of 12 on the assessed STROBE items in their results sections, highlighting the overall methodological rigor and transparency of the reported findings.

### Main genes

The exploration of the relationship between caesarean delivery and genetic factors impacting intelligence and anxiety traits in offspring revealed several significant genes identified through GWAS (Table 2). Key genes include FEM1AP3 and PA2G4P5 located at 6p12.3, whose functions remain elusive yet may influence neurodevelopment. The transcription factor LMX1A at 1q23.3 plays a crucial role in limb development and may also relate to intelligence through genetic pathways. MYLK2 on chromosome 20 is implicated in muscle contraction, suggesting a correlation between physical health and cognitive performance. TAF1A at 1q41 is essential for regulating gene expression, while LINC01502, a long non-coding RNA at 9q34.3, may affect gene expression relevant to intelligence and anxiety. The glutamate receptor gene GRIN2A at 16p13.2 underscores the importance of synaptic transmission in cognitive functions. Other regulatory genes, such as BACH2 (6q15) and DIP2C (10p15.3), are noteworthy, despite the latter’s unknown function. Moreover, CSGALNACT1 at 8p21.3 is associated with glycosylation processes impacting neuronal functions. The olfactory receptor genes OR4L1 and OR7H2P are linked to sensory pathways associated with anxiety traits, while ATXN1 (6p22.3) connects cognitive and physical genetic risks. Genes such as LRRFIP1 (2q37.3) and KRBOX1 (3p22.1) play pivotal roles in transcriptional regulation. Moreover, STMN4 and MIR548H4 at 8p21.2 are associated with critical microtubule dynamics in neurodevelopment, and long non-coding RNAs like LINC01924 and LINC01916 at 18q21.3 are emerging as potential regulatory elements. Lastly, CELSR1 at 22q13.31 emphasizes the importance of adhesion molecules in neuronal functions, highlighting the implications of genetic variations on cognitive traits.

**Table 2:** Significant genetic polymorphisms associated with intelligence and anxiety traits in offspring following Caesarean delivery.

**Risk Allele**	**Mapped Genes**	**Chromosome Location**	**Gene Function**	**p-Value**	**OR**	**CI**	**β**	**Trait in interaction with CS**
rs1276529-G	FEM1AP3, PA2G4P5	6p12.3	Unknown function	1E−6	-	0.22–2.86	2.04-unit decrease	Verbal intelligence score
rs1506946-A	LMX1A	1q23.3	Transcription factor involved in limb development	4E−6	-	1–2.45	1.73-unit decrease	Verbal intelligence score
rs6060973-T	MYLK2	20q11.21	Myosin light chain kinase, involved in muscle contraction	5E−6	-	1.9–4.75	3.33-unit decrease	Verbal intelligence score
rs17464857-G	TAF1A	1q41	Part of the transcription factor IID complex	6E−6	-	1.60–4.05	2.82-unit increase	Verbal intelligence score
rs705670-G	LINC01502	9q34.3	Long non-coding RNA, potential regulatory role	3E−7	-	1.42–3.18	2.3-unit increase	Performance intelligence score
rs4714020-T	C6ORF89	6p21.2	Epithelial cell growth; enhancing histone deacetylase; wound healing	7E−6	-	1.04–2.64	1.84-unit increase	Performance intelligence score
rs17800861-A	GRIN2A	16p13.2	Glutamate receptor, involved in synaptic transmission	7E−7	-	2.01–4.63	3.32-unit decrease	Overall intelligence score
rs9451316-T	BACH2	6q15	Transcriptional regulator, involved in B-cell development	7E−6	-	1.05–2.67	1.86-unit decrease	Overall intelligence score
rs10087146-C	CSGALNACT1	8p21.3	Involved in glycosylation processes	9E−6	-	1.4–3.6	2.5-unit decrease	Overall intelligence score
rs825885-A	TMEM132D, LINC02418	12q24.33	Regulate neuronal morphology	9E−6	-	1.16–3	2.08-unit decrease	Overall intelligence score
rs34416550-G	RNU6-168P, MAT2B	5q34	RNA component, involved in small nuclear RNA processing	6E−10	1.36	1.26–1.46	-	Anxiety
rs13137764-G	DKK2	4q25	Inhibitor of WNT signaling pathway	1E−9	1.47	1.34–1.6	-	Anxiety
rs11814503-T	DIP2C	10p15.3	Unknown function	1E−8	1.43	1.31–1.55	-	Anxiety
rs62522074-C	COL22A1	8q24.23-q24.3	Collagen type, involved in structural roles	1E−8	1.66	1.49–1.83	-	Anxiety
rs1959650-C	OR4L1, RNA5SP380	14q32.2	Olfactory receptor, potential sensory function	3E−8	1.29	1.2–1.38	-	Anxiety
chr10:565830-G	-	-	-	3E−8	1.44	1.31–1.57	-	Anxiety
rs62389045-C	ATXN1	6p22.3	Associated with spinocerebellar ataxia type 1	4E−8	2.17	1.89–2.45	-	Anxiety
rs62194228-A	LRRFIP1	2q37.3	Involved in the regulation of gene expression	6E−10	1.5	1.37–1.63	-	Self-harm
rs72933283-A	LINC01924, LINC01916	18q21.3	Long non-coding RNAs, potential regulatory roles	1E−9	2.53	2.23–2.83	-	Self-harm
rs189957265-T	LSAMP	3q13.31	Cell adhesion molecule, involved in neuronal function	2E−9	3.35	2.96–3.74	-	Self-harm
rs117077436-C	LINC02713, RNA5SP347	11q23.3	Long non-coding RNAs, potential regulatory roles	3E−9	11.34	10.54–12.14	-	Self-harm
rs144469617-A	LPL, RPL30P9	8p21.1	Lipoprotein lipase, involved in lipid metabolism	3E−9	2.44	2.14–2.74	-	Self-harm
rs80178526-T	OR7H2P, RNA5SP188	5q22.1	Olfactory receptor, potential sensory function	4E−9	1.7	1.52–1.88	-	Self-harm
rs77050423-G	MSRB3-AS1	12q24.31	Regulator of sulfur amino acid homeostasis	5E−9	2.76	2.42–3.1	-	Self-harm
rs183426309-C	KRBOX1, GASK1A	3p22.1	Transcription factor, involved in gene regulation	6E−9	3.36	2.95–3.77	-	Self-harm
rs567777268-T	LINC00348	13q32.2	Long non-coding RNA, potential regulatory role	9E−9	2.89	2.53–3.25	-	Self-harm
rs148128132-C	ST13P7, EXOC4	7q33	Involved in protein transport	1E−8	2.21	1.94–2.48	-	Self-harm
rs185846886-G	CDH9, CCNB3P1	5p14.1	Cadherin family member, involved in cell adhesion	1E−8	2.44	2.13–2.75	-	Self-harm
rs41276918-G	ANPEP	15q26.1	Aminopeptidase, involved in protein metabolism	1E−8	3.0	2.62–3.38	-	Self-harm
rs77828167-T	ALDH1A2	15q21.3	Aldehyde dehydrogenase, involved in retinoic acid metabolism	2E−8	3.11	2.72–3.5	-	Self-harm
rs75717648-A	STMN4, MIR548H4	8p21.2	Involved in microtubule dynamics	2E−8	1.92	1.69–2.15	-	Self-harm
rs115127002-G	TAGAP-AS1	6q24.2	Regulates T-cell activation	3E−8	2.56	2.23–2.89	-	Self-harm
rs72973905-A	COPS8-DT	2p16.1	Part of the COP9 signalosome complex	3E−8	2.65	2.31–2.99	-	Self-harm
rs116124269-T	DAB1	1p32.2-p32.1	Involved in signaling pathways during neuronal development	3E−8	2.0	1.75–2.25	-	Self-harm
rs140171389-AT	CELSR1	22q13.31	Cell adhesion molecule, involved in planar cell polarity	4E−8	5.05	4.47–5.63	-	Self-harm
rs75563143-C	COLEC12	18p11.32	Involved in immune response	4E−8	2.25	1.96–2.54	-	Self-harm

**Footnote:** Polymorphisms in the table are organized by descending P-value, with lower values indicating stronger association with traits linked to Caesarean delivery. OR (Odds Ratio) indicates the likelihood of a trait; OR > 1 suggests increased likelihood, while OR < 1 indicates a protective effect. CI (Confidence Interval) estimates the true effect size of the OR at 95 % confidence. Beta (β) reflects effect direction and magnitude: a positive β indicates increased trait score, while a negative β denotes decrease (e.g., β = −2.04 suggests reduced verbal intelligence score).

### SNP associations with intelligence, anxiety, and self-injury traits

**Fig. 1:**
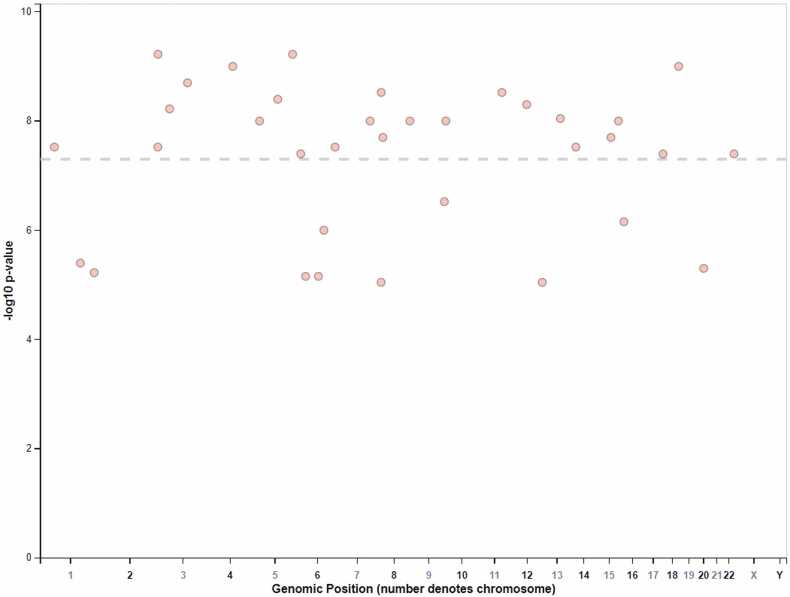
Distribution of SNPs associated with intelligence and anxiety traits in offspring, identified through GWAS in cesarean deliveries across the human genome.

Fig. 1 illustrates the genomic distribution of SNPs associated with intelligence and anxiety traits among offspring delivered by caesarean section. Table 2 provides a summary of key risk alleles, detailing their p-values, OR, CI, beta coefficients, related genes, chromosome locations, gene functions, and their associated intelligence traits.

1. Intelligence Related Traits
  Verbal Intelligence Score
  Four risk alleles adversely affect verbal intelligence scores. Notably, the rs1276529-G allele results in a significant decrease in verbal intelligence by 2.04 units (p = 1E-6) and is associated with the FEM1AP3 and PA2G4P5 genes on chromosome 6p12.3, whose functions remain unclear. The rs1506946-A allele is linked to the LMX1A gene on chromosome 1q23.3, presenting a decrease of 1.73 units (p = 4E-6) and is implicated in limb development. The rs6060973-T variant correlates with a 3.33-unit reduction (p = 5E-6) and is associated with the MYLK2 gene on chromosome 20q11.21, which is involved in muscle contraction. Conversely, the rs17464857-G allele is beneficial, enhancing verbal intelligence by 2.82 units (p = 6E-6) and is connected to the TAF1A gene on chromosome 1q41, part of the transcription factor IID complex.
  Performance Intelligence Score
  Two specific alleles have been identified as being associated with performance intelligence. The rs705670-G allele exhibits a significant correlation with an increase of 2.3 units (p = 3E-7) and is mapped to the LINC01502 locus on chromosome 9q34.3, which suggests a potential regulatory function through long non-coding RNA mechanisms. Similarly, the rs4714020-T variant shows a p-value of 7E-6 and is linked to a 1.84-unit increase in performance intelligence. This variant is associated with the C6ORF89 gene located on chromosome 6p21.2, which is implicated in epithelial cell growth and wound healing, therefore suggesting a genetic contribution to cognitive abilities.
  Overall Intelligence Score
  Four risk alleles related to overall intelligence scores have been identified, with three alleles correlating with decreases in intelligence. The rs17800861-A allele (p = 7E-7) is associated with a reduction of 3.32 units and is linked to the GRIN2A gene on chromosome 16p13.2, which encodes a glutamate receptor that is critical for synaptic transmission. Moreover, the rs9451316-T allele shows a 1.86-unit decrease (p = 7E-6) and relates to the BACH2 gene on chromosome 6q15, known for its role as a transcriptional regulator in B-cell development. The rs10087146-C variant (p = 9E-6) is associated with a reduction of 2.5 units and mapped to the CSGALNACT1 gene on chromosome 8p21.3, which is involved in glycosylation processes. Finally, the rs825885-A variant (p = 9E-6) correlates with a decrease of 2.08 units and is associated with TMEM132D and LINC02418 on chromosome 12q24.33, indicating a regulatory function in neuronal morphology.
2. **Anxiety**
  This study investigates the genetic factors influencing intelligence and anxiety traits in offspring born by caesarean delivery, utilizing GWAS data. Several risk alleles significantly associated with anxiety traits were identified. The rs34416550-G variant located on chromosome 5 (5q34) presents a p-value of 6E-10 and an OR of 1.36 (CI: 1.26–1.46), indicating a 36 % increased likelihood of developing anxiety. Additionally, the rs13137764-G allele on chromosome 4 (4q25) displayed a p-value of 1E-9 and an OR of 1.47 (CI: 1.34–1.6), suggesting a 47 % heightened risk. The rs11814503-T variant on chromosome 10 (10p15.3) showed a p-value of 1E-8 and an OR of 1.43 (CI: 1.31–1.55), reflecting a 43 % increased risk. Notably, the rs62522074-C variant on chromosome 8 (8q24.23-q24.3) had the highest OR of 1.66 (CI: 1.49–1.83) and a p-value of 1E-8, indicating a 66 % increase in anxiety risk. The rs1959650-C allele on chromosome 14 (14q32.2) had a p-value of 3E-8 and an OR of 1.29 (CI: 1.2–1.38), correlating to a 29 % higher likelihood of anxiety. The chr10:565830-G variant, which lacks specific gene mapping, showed a p-value of 3E-8 with an OR of 1.44 (CI: 1.31–1.57), suggesting a 44 % increased likelihood of anxiety. Finally, the rs62389045-C variant on chromosome 6 (6p22.3) exhibited a significant risk with a p-value of 4E-8 and an OR of 2.17 (CI: 1.89–2.45), indicating a substantial 117 % increase in anxiety risk, associated with the ATXN1 gene linked to spinocerebellar ataxia type 1.
3. **Self-Injury**

This study explores genetic factors associated with self-harm behaviors in offspring, particularly in the context of caesarean delivery, drawing upon GWAS data. Seventeen notable risk alleles were identified, with the most significant association found for the rs117077436-C variant (p-value: 3E-9; OR: 11.34), which connects to the long non-coding RNA LINC02713 and RNA5SP347 on chromosome 11q23.3. Another significant allele, rs189957265-T, has an OR of 3.35 (p-value: 2E-9) and is associated with the LSAMP gene located on chromosome 3q13.31, indicating its potential role in cell adhesion and neuronal functionality. Other prominent alleles include rs72933283-A (p-value: 1E-9, OR: 2.53) linked to long non-coding RNAs on chromosome 18q21.3, and rs77050423-G (p-value: 5E-9, OR: 2.76), associated with MSRB3-AS1, which is known to regulate sulfur amino acid homeostasis. Additional notable risk alleles such as rs567777268-T (p-value: 9E-9, OR: 2.89) and rs183426309-C (p-value: 6E-9, OR: 3.36) underscore the contributions of transcription factors and long non-coding RNAs in self-harm behaviors. Furthermore, rs144469617-A (OR: 2.44, p-value: 3E-9) indicates potential involvement in lipid metabolic pathways, while rs75563143-C (OR: 2.25, p-value: 4E-8) suggests an association with immune response mechanisms via the COLEC12 gene.

## Discussion

The interplay between genetic factors and delivery mode, particularly CS, significantly influences intelligence in offspring. Recent GWAS have identified ten genetic loci associated with intelligence, revealing how specific risk alleles affect cognitive performance. The SNP rs1276529-G is linked to an approximate 2.04-unit decrease in verbal intelligence scores (p = 1E-6), involving the genes FEM1AP3 and PA2G4P5, which require further research on their responses to delivery-related stress. Conversely, the rs17464857-G allele is associated with a 2.82-unit increase in verbal intelligence scores (p = 6E-6) and is connected to the TAF1A gene, suggesting some genetic factors may mitigate cognitive declines from Caesarean deliveries. Moreover, the SNP rs705670-G correlates with a 2.3-unit increase (p = 3E-7), pointing to cognitive benefits linked to the long non-coding RNA LINC01502. However, other alleles, such as rs17800861-A, show significant declines in intelligence (3.32-unit decrease, p = 7E-7), illustrating the complex effects of genetics on cognitive outcomes related to delivery methods. Genes like BACH2 and GRIN2A are significant for neurodevelopment in children born by CS [19]. BACH2, an immune-regulating transcription factor, may affect neurodevelopment through immune-mediated processes that promote neurogenesis and synaptic plasticity [20]. GRIN2A, which encodes a crucial NMDA receptor subunit for synaptic transmission and memory, shows variations linked to lower IQ scores in Caesarean-delivered children, particularly the SNP rs17800861 [14], [21], [22]. The relationship between genetic predispositions and environmental factors, such as gut microbiota—altered by delivery mode—further complicates our understanding of intelligence outcomes. Infants delivered by CS miss early exposure to maternal gut flora, vital for immune development and brain maturation, emphasizing the importance of genetics and environment in cognitive development [10].

Recent GWAS have also identified seven significant genetic loci—RNU6–168P, MAT2B, DKK2, DIP2C, COL22A1, OR4L1, and ATXN1—that influence anxiety traits in Caesarean-born offspring. Noteworthy variants like rs34416550-G and rs13137764-G, with ORs of 1.36 and 1.47 respectively, suggest increased anxiety risk. The allele rs62522074-C has the highest OR of 1.66, implicating COL22A1, associated with collagen structure, in anxiety development. Additionally, alleles rs1959650-C and chr10:565830-G show ORs of 1.29 and 1.44, indicating their roles in sensory and undefined anxiety pathways. The allele rs62389045-C, linked to ATXN1 and associated with neurological disorders, has an OR of 2.17. These findings underscore the complex interactions between genetic predispositions and environmental factors, such as CS, necessitating further investigation into their combined effects on anxiety. MAT2B is linked to anxiety behaviors through genetic variations impacting responses in animal models [23]. DKK2 plays a role in Wnt signaling, influencing neurodevelopment and synaptic plasticity, although its direct effects on anxiety remain unclear. DIP2C may affect emotional regulation but lacks definitive connections to anxiety. COL22A1 is crucial for connective tissue structure but has no direct anxiety associations; possible indirect influences on emotional health exist [24]. OR4L1, linked to olfactory functions regarding emotions, has yet to establish direct connections to anxiety disorders [25], [26]. Non-coding RNAs like RNU6–168P and RNA5SP380 may regulate stress and emotion, but definitive links to anxiety remain unconfirmed. ATXN1's role in transcription and neuronal functions suggests potential implications for emotional regulation, with its mutations possibly contributing to anxiety-like behaviors, especially in neurodegenerative contexts. Overall, while some genes, like MAT2B, show potential ties to anxiety, many require further exploration to understand the polygenic nature of anxiety disorders.

GWAS have identified 19 significant SNPs associated with self-harm in offspring delivered by Caesarean delivery, linked to genes such as LRRFIP1, LINC01924, LINC01916, and LSAMP. The SNP rs62194228-A exhibits a low p-value of 6E-10 and an OR of 1.5, indicating that carriers of this risk allele are 50 % more likely to self-harm than non-carriers. LRRFIP1 is crucial for gene expression regulation, significantly impacting emotional and behavioral outcomes. SNP rs72933283-A has a higher OR of 2.53, highlighting the importance of long non-coding RNAs in gene expression and psychiatric outcomes, especially among Caesarean-delivered offspring. The SNP rs189957265-T, with an OR of 3.35, is associated with the LSAMP gene, vital for brain development and neuronal function. In particular, rs117077436-C shows an OR of 11.34, necessitating further investigation into how long non-coding RNAs may modulate behavior under stressful birth conditions. Additional SNPs, like rs144469617-A (OR of 2.44, linked to lipid metabolism) and rs80178526-T (OR of 1.7, related to sensory functions), indicate complex genetic influences extending beyond psychiatric pathways to include metabolic and sensory systems affecting self-harm. SNPs rs77050423-G (OR of 2.76) and rs183426309-C (OR of 3.36) underscore regulatory roles during development that may be impacted by Caesarean delivery. These genes enhance our understanding of self-harm through genetic predispositions and psychiatric conditions [27], [28], [29]. For example, LRRFIP1 influences neuronal regulation and stress-related disorders, while lincRNAs such as LINC01924 and LINC01916 are associated with gene regulation and emotional behavior. LSAMP's role in neural development and stress responses may contribute to increased susceptibility to self-harm. Furthermore, lincRNAs like LINC02713 may correlate with mood disorders, and genes related to lipid metabolism, such as LPL, connect to mood regulation. Pseudogene RPL30P9 may influence neuronal health through protein synthesis, while OR7H2P, despite being an olfactory receptor pseudogene, might affect emotional states through sensory processing. Other relevant genes include MSRB3-AS1, linked to oxidative stress, and KRBOX1, involved in transcription regulation, alongside GASK1A, LINC00348, and ST13P7 related to neurodevelopment and stress. EXOC4 plays a role in neurotransmitter release, and CDH9 is essential for neural connectivity. The pseudogene CCNB3P1 may affect self-harm via neuronal health, and ANPEP influences neuropeptide metabolism linked with mood disorders [30], [31], [32]. Moreover, ALDH1A2 supports brain function through retinoic acid metabolism, while STMN4 contributes to neuronal plasticity and resilience. MicroRNA MIR548H4 regulates gene expression, potentially impacting mental health, and TAGAP-AS1 could moderate immune responses associated with self-harm. Moreover, COPS8-DT is tied to cellular stress responses, while DAB1 and CELSR1 are crucial for neuronal signaling and development. COLEC12's role in immune responses connects it to neuroinflammation and mental health issues, including depression and self-harm [33], [34].

### Conflicting evidence and limitations in current research

The interplay between Caesarean delivery and genetic influences on intelligence and anxiety traits in offspring has garnered considerable attention in recent research. Studies such as Zheng et al. (2022) suggest that children delivered via CS exhibit lower neurodevelopmental scores—covering gross motor, fine motor, and language development—compared to those born vaginally [7]. However, conflicting evidence indicates that CS may not significantly impact cognitive outcomes or could even confer advantages in specific contexts [35]. This discrepancy highlights the need for a nuanced understanding of how the mode of delivery interacts with various factors influencing child development, including mental health outcomes.

Research has explored the relationship between CS and mental health, with Lerche et al. reporting an 8 % increased risk of psychiatric admissions for individuals born via CS, suggesting a potential link to anxiety-related disorders [36]. Conversely, other studies have found no significant differences in anxiety or cognitive outcomes based on delivery method [37]. This variability underscores the complexity of the relationship between delivery mode and mental health, as the impact of CS may be moderated by other factors, such as the infant's environment and genetic predispositions. Particularly, the role of gene-environment interactions in understanding anxiety risks is critical, with genetic variants like DKK2 and ATXN1 showing interactions with CS [17]. It is essential to recognize that not all genetic influences lead to adverse outcomes; some individuals may possess protective genetic factors that mitigate the risks associated with CS. Parental genetics can shape offspring cognition indirectly through environmental effects, such as parental education and socioeconomic status [38]. Therefore, while the mode of delivery is an environmental factor that may influence cognitive and mental health outcomes, it is only one among many factors at play, including the mechanisms of epigenetic programming and microbiome transfer.

The hormonal fluctuations and stress responses associated with vaginal deliveries may induce epigenetic changes that affect gene expression and neurodevelopment—changes that may not occur in CS births [39], [40]. Nevertheless, not all research supports the notion that CS disrupts beneficial microbiome transfer or leads to negative outcomes; some studies suggest that CS infants can adapt well, particularly with appropriate postnatal care and interventions [41]. Understanding the intricate relationship between genetic and environmental factors, including the mode of delivery, has significant implications for targeted interventions aimed at alleviating the risks associated with Caesarean deliveries. Thus, potential strategies, such as probiotics or microbiome seeding, could help mitigate long-term health outcomes linked to CS [39]. Moreover, genetic counseling and personalized medicine may play critical roles in identifying individuals at elevated risk for anxiety or cognitive delays based on their genetic predispositions and birth histories [17]. A balanced perspective that encompasses both the potential risks and benefits of CS is essential for advancing our understanding of its impact on child development.

### Confounding factors influencing cognitive and emotional development in Caesarean-born children

The relationship between CS and cognitive and emotional development in offspring is influenced by a complex array of confounding factors, including genetic and environmental variables. Observational studies by Blake et al. [8] and Hanrahan et al. [42] have indicated that children born via CS may experience deficits in cognitive performance, particularly in visual-spatial skills. Further evidence from Xu et al. (2023) suggests that these children exhibit lower performance IQ scores and heightened ADHD indices [16]. However, the apparent associations may be confounded by parental traits and environmental factors. For instance, Smajlagic et al. [14] found no genome-wide significant interactions between CS and genetic variants associated with intelligence, indicating the possible influence of socioeconomic status and parental intelligence on cognitive outcomes, a notion supported by Meireles and Machado, who reported that controlling for these factors could attenuate the CS-cognitive outcome association [43].

Anxiety disorders present another dimension to the effects of CS, as several studies have shown an increased risk of anxiety and mental health issues among individuals delivered by CS. Jia et al. [17] utilized UK Biobank data to reveal increased odds of anxiety (OR = 1.25) and self-harm (OR = 1.18) among this cohort. Complementary findings from a Finnish birth cohort study further validate that both planned and unplanned CS are correlated with elevated risks of anxiety disorders in children and adolescents [17]. The interplay of genetic and environmental confounding factors is critical, as demonstrated in recent studies. For example, Thapaliya et al. (2024) noted that specific genes (e.g., DKK2 and ATXN1) interact with CS to influence anxiety outcomes, while societal factors like early life stress and socioeconomic status have also emerged as significant predictors of anxiety traits across populations [44].

Socioeconomic status serves as a crucial confounder in understanding the relationship between CS and offspring outcomes. Research, such as that from the Adolescent Brain and Cognitive Development Study (2023), indicates that lower familial and neighborhood socioeconomic status heightens the risks of anxiety and psychological issues in children [45]. Conversely, a Hong Kong-based cohort study revealed an association between CS and higher socioeconomic status, complicating the interpretation of psychological well-being outcomes related to delivery mode [46]. Additional confounding factors include prenatal conditions, as evidence from Chen et al. (2023) suggests that interactions between high birth weight and emergency CS elevate ADHD risks [47]. While some research points to potential direct effects of CS on genetic pathways and long-term outcomes [48], the prevailing viewpoint suggests that observed differences primarily reflect pre-existing parental traits rather than the delivery method itself. This concept is further supported by Dachew et al. (2022) [49] and Lerche et al. [36], who argue that the increased risks associated with CS may derive more from unmeasured confounding factors rather than direct causal pathways.

### Clinical implications

Recent GWAS findings underscore critical clinical implications for healthcare providers regarding the cognitive and emotional development of children born via CS. It is essential to consider the interaction between genetic predispositions and environmental factors in assessing these children's development. Clinicians should closely monitor their cognitive performance and emotional health, particularly for those identified with risk factors linked to specific genetic loci associated with intelligence, anxiety, and self-harm. To address these implications, future research should focus on longitudinal studies that track the developmental trajectories of children born via CS, particularly in relation to their genetic profiles. Additionally, investigating the effectiveness of early interventions, such as integrated psychological support and developmental monitoring, could provide valuable insights into mitigating potential adverse effects. Clinicians should prioritize educating parents about the potential impact of CS on development. Promoting socioeconomic stability and addressing environmental stressors will be crucial in improving outcomes for these children. Furthermore, incorporating genetic screening linked to family history may facilitate the development of tailored support and intervention strategies. A comprehensive approach that integrates ongoing research, clinical practice, and family education is necessary to adequately address the developmental and mental health needs of children born via CS.

### A Cautious Interpretation of results

In discussing the genetic associations identified in this study, it is imperative to address the concerns regarding their interpretation, particularly given that numerous single nucleotide polymorphisms (SNPs) associated with intelligence and anxiety traits in offspring born via caesarean delivery exhibit relatively small effect sizes. These minor impacts should be contextualized within the broader framework of genetic influences, as they do not serve as definitive predictors of cognitive or anxiety-related outcomes. The biological significance of the identified SNPs remains largely unclear—for instance, while associations with olfactory receptor genes like OR4L1 and OR7H2P suggest links to sensory pathways potentially related to anxiety traits, the mechanistic pathways through which these alleles operate are still speculative. Furthermore, genes such as FEM1AP3 and PA2G4P5 at 6p12.3, whose functions are not well understood, necessitate cautious interpretation regarding their relationship with intelligence. Similarly, genes involved in muscle contraction (MYLK2) and neuronal function (GRIN2A and CSGALNACT1) hint at potential connections between physical health and cognitive performance; however, the precise mechanisms by which these genes might influence such traits require further investigation. Given the small effect sizes commonly observed in GWAS findings, future research must focus on uncovering the molecular underpinnings of these SNPs to elucidate their mechanistic relevance to intelligence and anxiety. Addressing these complexities and promoting careful interpretation will foster a balanced understanding of the genetic landscape governing these traits. Thus, while the associations presented in this study contribute valuable insights into the genetic determinants of intelligence and anxiety, they should be viewed as preliminary findings that establish a foundation for more in-depth explorations.

### Advantages and limitations

The review article offers a comprehensive examination of GWAS exploring the links between CS and traits like intelligence and anxiety. Its main strength lies in synthesizing findings from multiple studies, enhancing our understanding of genetic influences across different ancestral backgrounds. The clear presentation of significant genetic variants and statistical measures aids in comprehending the genetic factors related to these traits. Additionally, the interdisciplinary approach connecting genetics, psychology, and obstetrics may inspire new hypotheses about the biological mechanisms behind intelligence and anxiety and the potential effects of delivery methods on offspring traits. However, the review notes several limitations. It analyzes only five GWAS studies, with two by the same author, raising concerns about selection bias and the generalizability of the results. Potential confounding factors, such as socioeconomic status and parental intelligence, may not be adequately addressed, possibly skewing the observed associations. Variability in sample sizes across studies could also undermine the robustness of the findings, especially given the diverse methods for measuring intelligence and anxiety. While the participant base is diverse, the predominance of individuals of European descent may limit the findings' relevance to non-European populations. The complexities of intelligence and anxiety, shaped by various genetic and environmental influences, warrant careful consideration. A narrow focus on specific genetic variants risks oversimplifying these complexities and neglecting significant gene-environment interactions. Finally, the small effect sizes of GWAS associations highlight the need for cautious interpretation, as they may indicate correlations rather than strong causal relationships.

## Conclusions

In summary, this analysis underscores the complex relationship between genetic factors and CS concerning variations in intelligence and anxiety levels among offspring. While certain risk alleles may be linked to cognitive abilities and delivery-related vulnerabilities, the study's limitations must be acknowledged. The reliance on associative data restricts causal conclusions, and factors like socioeconomic status and parental intelligence were insufficiently controlled, raising questions about the generalizability of the findings, particularly given the predominance of participants of European descent. Future research should prioritize gene-environment interactions to clarify how delivery methods and early experiences might influence genetic impacts on cognitive and psychiatric traits. Longitudinal studies involving diverse populations will be crucial for validating these associations. Furthermore, further exploration of the biological mechanisms behind these connections is necessary. Although this review recognizes the potential significance of the interactions studied, it refrains from making broad clinical or policy recommendations and stresses the need for further research before drawing definitive conclusions.

## Ethics approval

This article does not involve studies with human or animal participants conducted by the authors.

## Consent to participate

Not applicable.

## Consent for publication

Not applicable.

## Funding

No funding source was provided.

## CRediT authorship contribution statement

**Rashnavadi Heewa:** Software. **Jayervand Fatemeh:** Methodology, Investigation. **Azizi Sepideh:** Methodology. **Dastgheib Seyed Alireza:** Data curation, Conceptualization. **Bahrami Reza:** Validation. **Shahbazi Amirhossein:** Writing – review & editing, Writing – original draft. **Masoudi Ali:** Software. **Aghili Kazem:** Data curation. **Nematzadeh Fatemeh:** Writing – review & editing, Writing – original draft. **Danaei Mahsa:** Conceptualization. **Neamatzadeh Hossein:** Writing – review & editing, Writing – original draft, Supervision, Conceptualization. **Fallahpour Bita:** Conceptualization. **Yeganegi Maryam:** Conceptualization.

## Declaration of Competing Interest

The authors declare that they have no known competing financial interests or personal relationships that could have appeared to influence the work reported in this paper.

## Data Availability

Datasets generated and analyzed in this study are available from the corresponding author upon reasonable request.
